# Prävention und Therapie langfristiger Beschwerden kritisch erkrankter Patientinnen und Patienten – Verantwortung der Intensivmedizin

**DOI:** 10.1007/s00101-025-01597-4

**Published:** 2025-09-30

**Authors:** Mahan Sadjadi

**Affiliations:** Klinik für Anästhesiologie, operative Intensivmedizin und Schmerztherapie, Universitätsklinikum Münster, Albert-Schweitzer-Campus 1, Gebäude A1, 48149 Münster, Deutschland

**Keywords:** Post-Intensive Care Syndrom, PICS, Kritische Erkrankung, Organdysfunktion, Nachsorge, Post-Intensive Care Syndrome, PICS, Critical illness, Organ dysfunction, Long-term outcome

## Abstract

**Hintergrund:**

Patient:innen, die eine kritische Erkrankung mit intensivmedizinischer Behandlung überstanden haben, leiden häufig unter anhaltenden Beschwerden, die als Post-Intensive Care Syndrom (PICS) zusammengefasst werden. PICS umfasst sowohl neu aufgetretene als auch sich verschlechternde Symptome, die die Lebensqualität und Alltagsfunktionalität einschränken und so die gesellschaftliche und berufliche Reintegration erschweren. Um dem vorzubeugen, gewinnen neben der Verbesserung der Akuttherapie auch die Übergänge von der Intensiv- zur Regelversorgung sowie die langfristige Betreuung an Bedeutung.

**Ziel der Arbeit:**

Relevante Risikofaktoren sowie präventive und therapeutische Maßnahmen für die langfristige Versorgung kritisch erkrankter Patient:innen werden identifiziert und diskutiert. Der Fokus liegt auf der Rolle der Intensivmedizin in der Weichenstellung für eine optimale Nachsorge.

**Methoden:**

Auf Grundlage einer systematischen Literaturrecherche werden die Ergebnisse bisheriger Studien narrativ synthetisiert, um Implikationen für die wissenschaftliche und klinische Praxis abzuleiten.

**Ergebnisse und Diskussion:**

Bis zu 70 % kritisch erkrankter Patient:innen leiden im ersten Jahr nach der Intensivtherapie an relevanten Einschränkungen. Neben der Optimierung der Akuttherapie in Abhängigkeit von individuellen Bedarfen sind die systematische Dokumentation und verlustfreie Kommunikation des intensivstationären Verlaufs sowie eine enge interdisziplinäre Zusammenarbeit essenziell, um langfristige Komplikationen zu minimieren. Die Intensivmedizin spielt dabei eine Schlüsselrolle, indem sie sicherstellt, dass durch eine bestmögliche Weichenstellung während der Akutversorgung individuelle kurz-, mittel- und langfristige Ziele zeitgerecht erreicht werden können.

## Abstract (en)

**Background:**

Patients who have survived a critical illness requiring intensive care treatment often suffer from persistent complaints, collectively referred to as post-intensive care syndrome (PICS). This syndrome includes both new and worsening symptoms that impair the quality of life and restrict activities of daily living and often hinder successful social and occupational reintegration. In addition to improving acute treatment, the transition from the intensive care unit (ICU) to standard care and long-term follow-up are rapidly becoming an important area of care improvement.

**Objective:**

This review identifies and discusses relevant risk factors as well as preventive and therapeutic measures for the long-term care of critically ill patients. The focus is on the role of intensive care medicine in setting the course for optimal follow-up after critical illness.

**Methods:**

Based on a systematic literature search, the results of previous studies are narratively synthesized to derive implications for the scientific and clinical practice.

**Results and discussion:**

Up to 70% of critically ill patients experience relevant impairments during the first year after intensive care treatment. Besides optimization of acute treatment depending on individual needs, the systematic documentation and seamless communication of information regarding the intensive care course as well as close interdisciplinary collaboration are essential to minimize long-term complications. Intensive care medicine plays a key role in the care of critically ill patients even beyond ICU discharge by ensuring that, through sound decision-making and planning of the next steps during intensive care, short-term, medium-term and long-term goals can be achieved in a timely manner.

## Einführung

Wenn Patientinnen und Patienten die Intensivstation verlassen, haben sie die kritischste Phase ihrer Erkrankung überstanden. Der Prozess der Genesung ist mit der Entlassung aber keineswegs abgeschlossen. Vielmehr beginnt für viele Patient:innen erst nach der Verlegung von der Intensivstation ein bewusst erlebter, oft langwieriger und von unvorhergesehenen Herausforderungen begleiteter Teil ihrer Rekonvaleszenz, der sich über Wochen, Monate und Jahre erstrecken kann. Das Ziel dieser Arbeit ist es, zu diskutieren, welche Rolle die Intensivmedizin einnehmen kann, um individuelle Risiken früh zu erkennen, präventive Maßnahmen einzuleiten und dafür zu sorgen, dass nicht nur akutes, sondern auch langfristiges Leid gelindert wird.

### Klinisches Bild und Prävalenz

Anhaltende Beschwerden nach überstandener kritischer Erkrankung werden unter dem Begriff Post-Intensive Care Syndrom (PICS) zusammengefasst. Der Begriff umfasst neue, im Zusammenhang mit einem Intensivstationsaufenthalt entstandene körperliche, kognitive und psychische Symptome ebenso wie das Fortschreiten präexistenter Einschränkungen. Diese Beschwerden können unmittelbar nach der Entlassung oder auch erst verzögert auftreten, betreffen häufig mehrere Dimensionen gleichzeitig und stellen für Betroffene wie auch für deren Angehörige eine erhebliche Einschränkung der Lebensqualität dar. Ergänzend zum PICS sind spezifische Organdysfunktionen und ihre Persistenz nach der Akutphase der Erkrankung zu berücksichtigen, wobei beide Phänomene in einem engen Verhältnis zueinander stehen und sich teils gegenseitig bedingen [1–5]. Das Risiko für das Auftreten langfristiger Beeinträchtigungen wird durch zahlreiche Faktoren beeinflusst, darunter der funktionelle und psychische Status vor Aufnahme auf die Intensivstation, bestehende Komorbiditäten, Art und Schwere der akuten Erkrankung und Intensität der notwendigen Therapie. Je nach Patient:innenkollektiv berichten bis zu 70 % der Überlebenden im ersten Jahr nach der Entlassung von der Intensivstation über persistierende, alltagsrelevante Beschwerden [6–8].

Zu den körperlichen Symptomen gehören Muskelschwäche und schnelle Ermüdbarkeit, die zu einer deutlichen Beschränkung der Aktivitäten des täglichen Lebens (Activities of Daily Living, ADL) führen [9]. Kognitive Einschränkungen äußern sich als Reduktion von Gedächtnis- und Konzentrationsleistung, und psychische Symptome manifestieren sich in Form von Depressionen, Angststörungen oder posttraumatischen Belastungsstörungen. In vielen Fällen sind die Beschwerden so schwerwiegend, dass die Rückkehr in das Berufsleben und das Wiedererlangen sozialer und ökonomischer Integration erheblich erschwert werden [1, 3, 4, 10, 11].

## Organdysfunktionen, Therapiemaßnahmen und ihre Langzeitfolgen

Viele intensivmedizinische Krankheitsbilder sind komplex und verlaufen systemisch und somit organübergreifend. Im Lichte dessen – und angesichts der hohen Prävalenz bleibender Symptome – drängt sich der Schluss auf, dass die Therapie mit der Beendigung der Akutmaßnahmen (also der unmittelbaren Maßnahmen auf der Intensivstation) noch nicht abgeschlossen sein kann. Akut auftretende Organdysfunktionen wie die akute Nierenschädigung (Acute Kidney Injury, AKI), das Acute Respiratory Distress Syndrome (ARDS) oder Syndrome wie die Sepsis, aber auch alle therapeutischen Gegenmaßnahmen, sind nicht nur in Bezug auf sofortige Implikationen, sondern auch im Hinblick auf ihre langfristigen Auswirkungen zu berücksichtigen. So können noch während der Akuttherapie die Übergänge von der akuten zur chronischen Phase beziehungsweise einer Folgeerkrankung mit Abnahme der Organreserve mitgedacht und die Sekundärprävention vorbereitet werden.

### Akute Organdysfunktion am Beispiel der AKI

Die AKI ist ein klassisches Beispiel eines akuten Krankheitsbildes mit einem durch Abnahme der Organfunktion und -reserve begründeten potenziellen Übergang in eine chronische Organdysfunktion. Mit einer Inzidenz bis zu 66 % ist sie gleichzeitig eine der häufigsten Komplikationen kritisch kranker Patient:innen [12, 13]. Eine (anhaltende) Reduktion der Nierenfunktion beeinflusst verschiedene Organsysteme, allen voran das Herz-Kreislauf-System, und ist ein wichtiger Risikofaktor für die Entwicklung einer dialysepflichtigen chronischen Nierenerkrankung [12–14]. Um diesen gravierenden Langzeitfolgen vorzubeugen, muss eine AKI während der Intensivtherapie früh erkannt, ihr Progress nach Möglichkeit verhindert und ihr Verlauf genau dokumentiert werden, um im Anschluss eine adäquate Weiterversorgung und Optimierung der Therapie organisieren zu können. In der Akutversorgung bedeutet das z. B. die Implementierung eines Maßnahmenpakets zur Nephroprotektion, basierend auf den Leitlinien der Kidney Disease: Improving Global Outcomes (KDIGO), welche u. a. eine strukturierte hämodynamische Optimierung und die Vermeidung nephrotoxischer Medikamente vorsehen [15–17]. Notwendig ist aber auch die genaue Dokumentation des Schweregrads und Verlaufs der akuten Organschädigung, die zentrale Informationen für die Nachsorge darstellen. Für die Weiterversorgung ist dann eine Überprüfung der Nierenfunktionsparameter nach 3 Monaten zu planen. Wenn darauf basierend die Diagnose einer chronischen Nierenfunktionseinschränkung gestellt wird, gehören dazu auch die Einleitung einer medikamentösen Sekundärprävention sowie die strenge Einstellung anderer Risikofaktoren wie einer arteriellen Hypertonie und eines Diabetes mellitus, um einem Erkrankungsprogress vorzubeugen [14, 18]. Dass das tatsächlich passiert, ist, soweit sich das anhand der verfügbaren Datenlage abschätzen lässt, die Ausnahme: Das Problem beginnt damit, dass eine aufgetretene renale Dysfunktion, ihre Ursachen und ihr Verlauf in weniger als der Hälfte der Entlassungsberichte auftauchen und weniger als 10 % der Briefe Empfehlungen für die Weiterversorgung enthalten [14, 18, 19]. Bei nur einem Viertel aller Patient:innen mit überstandener AKI erfolgen 3 Monate nach der Entlassung eine Untersuchung des Urins und eine Bestimmung des Serumkreatininwerts [19, 20]. Die allerwenigsten Patient:innen wissen überhaupt, dass sie eine Nierenschädigung erlitten haben, was verhindert, dass sie aus eigenem Antrieb aktiv werden können [21, 22]. Gerade unter dem Gesichtspunkt, dass solche Herausforderungen für sämtliche intensivmedizinischen Krankheitsbilder zutreffen können, ist es letztlich an der Intensivmedizin, gegenzusteuern und eine nahtlose Anschlussversorgung zumindest anzuregen.

### Einschränkungen durch Therapiemaßnahmen am Beispiel des ARDS

Neben der eigentlichen Erkrankung kann sich auch die während des Intensivstationsaufenthaltes notwendige Therapie auf Outcomes auswirken, was anhand des Beispiels prolongierter Beatmung deutlich wird. Obwohl dank moderner Intensivmedizin immer mehr Patient:innen die akute Phase von Erkrankungsbildern wie dem ARDS überstehen, können Komplikationen der invasiven Behandlung erhebliche Langzeitfolgen nach sich ziehen. Neben pulmonalen Einschränkungen kann die beim ARDS notwendige Beatmungstherapie zu einer postintensivmedizinischen Muskelatrophie führen; diese betrifft insbesondere (aber nicht nur) die Atemmuskulatur und kann die Rehabilitation und Rückkehr zu gewohnten Aktivitäten erschweren. Hiervon ist etwa ein Drittel der Überlebenden in schwerer Ausprägung betroffen [23–27]. Neben diesen physischen Folgen leiden Betroffene häufig an psychischen und kognitiven Einschränkungen. Bei Entlassung aus dem Krankenhaus betrifft das fast jeden einzelnen Patienten und jede Patientin (70–100 %) [23, 28, 29]. Ein Jahr später leiden noch immer rund 80 % der Überlebenden an solchen Symptomen, was anhand publizierter Daten einerseits aus dem erlebten Stress der Therapie und andererseits aus der dem Erkrankungsbild zugrunde liegenden Pathophysiologie resultiert [23, 28, 29]. Die Behandlung dieser bleibenden Nachwirkungen erfordert eine entsprechende Expertise [24, 26, 28, 30]. Neben der Vorbeugung und leitliniengerechten Therapie eines ARDS sollte bei Auftreten schon aus der Intensivmedizin heraus angeregt werden, dass nicht nur die Lungenfunktion kontrolliert wird, sondern bei prolongiertem Intensivstationsaufenthalt auch frühzeitig physio- und ergotherapeutische sowie ggf. auch psychotherapeutische Maßnahmen angedacht werden, um eine Rehospitalisation sowie Morbidität und Mortalität zu vermeiden [25, 31, 32].

### Langzeitfolgen intensivmedizinischer Syndrome: die Sepsis

Besonders wichtig ist ein solches Konzept zur frühzeitigen Organisation strukturierter Weiterversorgung bei komplexen intensivmedizinischen Syndromen wie der Sepsis. Die akute Phase der Erkrankung geht mit einer hohen Mortalität einher. Überleben Betroffene diesen akuten Verlauf, leiden sie überdurchschnittlich häufig unter persistierenden Organdysfunktionen und PICS-Symptomen (bis zu 70 %) [33–41]. Funktionelle Langzeitfolgen nach einer Sepsis werden aus diesem Grund auch als Post-Sepsis-Syndrom beschrieben, wobei starke konzeptionelle Überlappungen zum Post-Intensive Care Syndrom zu erkennen sind [42]. Der Zusammenhang zwischen Sepsis und kognitiver Dysfunktion (vermutlich begründet durch Mikrozirkulationsstörungen sowie inflammatorische Prozesse und deren direkte Auswirkungen auf das Gehirn) steht im Mittelpunkt der langfristigen klinischen Versorgung Betroffener [43, 44]. Diese neurologischen Langzeitoutcomes sind für Patient:innen und deren Angehörige eine der größten Sorgen und werden maßgeblich durch das Auftreten und die Schwere eines Delirs beeinflusst, das ebenso wie die postseptische Enzephalopathie, zu der viele Überschneidungen bestehen, das Risiko für neurodegenerative Symptome – insbesondere anhaltende kognitive Beeinträchtigungen und Gedächtnisstörungen – erhöht [45–48]. Diesem Umstand ist die prominente Positionierung nichtmedikamentöser Delirprävention in deutschen und amerikanischen Leitlinien geschuldet [49, 50]. Nichtsdestotrotz leidet, je nach Aufnahmediagnose und intensivstationärem Verlauf nahezu jeder zweite Betroffene noch Monate nach Abschluss der Therapie unter kognitiven Einschränkungen [47, 51–58]. Überlebende einer Sepsis schildern eine durch diese Nachwirkungen so nachhaltig eingeschränkte Lebensqualität, dass die Prävention einer solchen Symptomatik als zentrales Ziel der Therapie im Sinne einer Optimierung patientenzentrierter Outcomes betrachtet werden muss. Diese Patientengruppe kann deshalb möglicherweise auch in besonderem Maße von der standardisierten Implementierung einer Langzeittherapie profitieren [33–37].

## Status quo der Risikoerfassung und Weichenstellung

Die strukturierte Erfassung individueller Risikofaktoren wie z. B. des funktionellen Status *vor* der Aufnahme auf die Intensivstation – eines der stärksten Prädiktoren für die Auftretenswahrscheinlichkeit und das Ausmaß späterer Einschränkungen – stellt aktuell noch die Ausnahme dar [59–61]. Ein fehlender Überblick über den Zustand des Patienten oder der Patientin vor der kritischen Erkrankung und später das Fehlen einer akkuraten Dokumentation des Intensivstationsaufenthaltes erschweren die Risikoabschätzung sowie die Entwicklung individualisierter Präventionsstrategien. So werden Beschwerden nach der Entlassung verspätet erkannt oder nicht adäquat zugeordnet [62]. Diskontinuitäten im Informationsfluss zwischen Intensivstation und nachfolgenden Versorgungsstufen führen dazu, dass sowohl Patient:innen als auch weiterbehandelnde Ärzt:innen die Verbindung zwischen aktuellen Problemen und dem intensivmedizinischen Verlauf nicht erkennen. Zahlreiche Betroffene wenden sich deshalb verspätet ärztlicher Hilfe zu, was es für Haus- und Fachärzt:innen schwierig macht, diffuse Beschwerden wie Konzentrationsstörungen, Muskelschwäche oder psychische Belastung auf einen teils länger zurückliegenden Intensivstationsaufenthalt zurückzuführen [63–71]. Hierdurch kommen neue, validierte Erhebungsinstrumente wie der deutschsprachige PICS-Fragebogen oder andere Screening-Tools im ambulanten Setting seltener zum Einsatz, als es möglich wäre. Ohne zügige Diagnostik verstreicht so wertvolle Zeit, in der eine frühzeitige Rehabilitation besonders effektiv sein könnte [72–74].

## Chancen und Verantwortung der Intensivmedizin

**Abb. 1:**
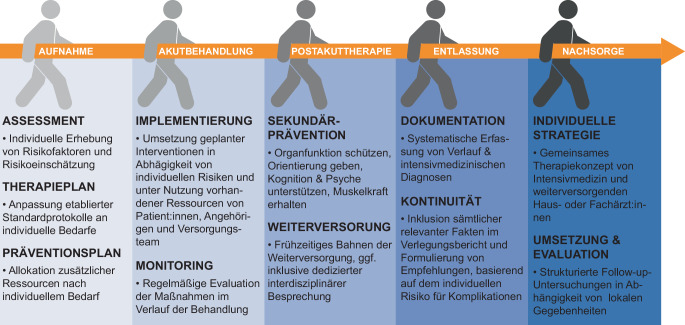
Vorbeugende Maßnahmen als Beitrag der Intensivmedizin zu Verbesserung von Langzeitoutcomes von kritisch erkrankten Patientinnen und Patienten

Intensivmediziner:innen können zu unterschiedlichen Zeitpunkten eine Einschätzung bezüglich langfristiger Risiken für ihre Patientinnen und Patienten treffen und daraus Konsequenzen ableiten. So können einerseits während der Intensivtherapie zielgerichtet präventive Maßnahmen ergriffen werden, und andererseits kann so die Anschlusstherapie frühzeitig gebahnt werden. Konkrete Handlungsempfehlungen sind, nach dem jeweiligen Zeitpunkt im Behandlungsablauf strukturiert, in Abb. 1 zusammengefasst.

### Aufnahme auf die Intensivstation: Risikoeinschätzung und präventive Maßnahmen

Die Identifikation von Patient:innen mit Risiken sollte schon bei Aufnahme auf die Intensivstation erfolgen. Hierzu gehört, wie bereits auf den allermeisten Intensivstationen Standard ist, die strukturierte Erfassung etwaiger Vorerkrankungen und Vormedikationen.

Neben somatischen Grunderkrankungen und den Aufnahmediagnosen stehen hinsichtlich der psychischen Gesundheit Depressionen, Angststörungen oder posttraumatische Belastungsstörungen (PTBS) im Vordergrund. Vorbestehende Einschränkungen der psychischen Gesundheit können durch den Stress intensivmedizinischer Versorgung aggraviert werden, was den Genesungsprozess verzögert. Hier können frühzeitige Maßnahmen, wie psychiatrisch-psychosomatische Konsultationen, möglicherweise helfen, spätere Komplikationen zu minimieren [6–8].

Auch kognitive Einschränkungen sollten berücksichtigt werden. Patient:innen, die bereits vor der Intensivbehandlung kognitive Beeinträchtigungen oder neurodegenerative Erkrankungen wie eine Demenz hatten, sind anfälliger für kognitive Funktionsstörungen während der Intensivtherapie und nach der Entlassung [28, 75–77]. Eine prästationäre Erhebung von Baseline-Daten zu kognitiven Fähigkeiten kann helfen, die Herangehensweise zu individualisieren. Für Patient:innen, die ungeplant aufgenommen werden und bei denen ein direktes Erheben von Scores nicht möglich ist, z. B. aufgrund einer erkrankungsbedingten Veränderung des Bewusstseins oder iatrogener Sedierung, kann eine etwaig vorhandene Dokumentation genutzt oder ersatzweise Angehörige konsultiert werden. Während des Aufenthalts können diesen Patient:innen so Ressourcen, die dazu beitragen, einem Erkrankungsprogress oder dem Auftreten von Komplikationen vorzubeugen, zur Verfügung gestellt werden.

Die Hausmedikation, die in der Regel zumindest teilweise pausiert wird, sollte dokumentiert, sorgfältig evaluiert und zeitnah wieder aufgenommen oder angepasst werden [78, 79].

Letztlich steht naturgemäß der Schweregrad der Akuterkrankung im Zentrum der zielgerichteten Therapie. Er bietet auch einen der relevantesten Anhaltspunkte für das Abschätzen langfristiger Risiken [7, 80]: Patient:innen mit schwerem akuten Organversagen haben ein höheres Risiko für Langzeitkomplikationen [23, 39, 45, 51, 81]. Neben der unmittelbaren Behandlung ist es daher wichtig, den Schweregrad der Erkrankung und etwaiger Komplikationen während der Intensivtherapie genau zu erfassen, damit weiterbehandelnde Ärzt:innen und andere Gesundheitsfachkräfte ein vollständiges Bild des Verlaufs gewinnen und die weitere Therapie individuell anpassen können.

### Individualisierte Therapie auf der Intensivstation

Während der Behandlung auf der Intensivstation verdient der Erhalt der Organfunktion, wie oben beschrieben, zentrale Aufmerksamkeit. Unterstützend können aber verschiedene weitere interdisziplinäre Maßnahmen dazu beitragen, langfristige Outcomes zu optimieren. Dazu kann z. B. die systematische physiotherapeutische und ergotherapeutische Behandlung gehören, um physische Ressourcen der Patient:innen zu erhalten, auch wenn die Evidenzlage hierbei dadurch begrenzt ist, dass kein über die Entlassung hinaus anhaltender Effekt gezeigt werden konnte [82–85]. Auch eine frühe psychologische Betreuung kann für ausgewählte Patient:innen einen Beitrag dazu leisten, der Entwicklung oder Verschärfung von Angststörungen, Depressionen und posttraumatischen Belastungsstörungen vorzubeugen [86]. Auf dieser Grundlage sollte bereits in der frühen Postakutphase das Fundament für eine erfolgreiche Langzeitrehabilitation gelegt werden. Es empfiehlt sich, Angehörige, wenn vorhanden, früh in den Behandlungsprozess einzubeziehen – nicht nur mit der passiven Tätigkeit des „Informiertwerdens“, sondern, je nach individuellen Ressourcen, als Partner in der langfristigen Gewährleistung optimaler Versorgung [87–89]. Dazu gehört neben dem Begreifbarmachen des Erkrankungsverlaufs z. B. das Angebot, Intensivtagebücher zu führen, um in späteren Phasen, spätestens in der ambulanten Nachsorge, mit einem umfassenden Verständnis aktiv werden und unterstützen zu können. Speziell für Intensivtagebücher konnte sehr robust gezeigt werden, dass ihr Einsatz die Inzidenz psychischer Symptome nach der Entlassung reduzieren kann, sodass ihre flächendeckende Implementierung angestrebt werden sollte [90–92].

Der Fokus der intensivmedizinischen Bemühungen liegt je nach Verlauf der Erkrankung und des ICU-Aufenthalts jeweils auf unterschiedlichen Aspekten. Was für langfristige Outcomes zählt, ist, dass je nach Phase diejenigen Maßnahmen getroffen werden, von denen ein positiver langfristiger Effekt erwartet werden kann (Abb. 1).

Die strukturierte Implementierung von Maßnahmen und deren Vergütung bedürfen einer soliden Datengrundlage, die in den kommenden Jahren erarbeitet werden muss. Klar ist aber auch, dass das Adressieren des Leids Betroffener nicht auf eine vollständige Konsolidierung wissenschaftlicher und klinischer Definitionen warten kann. Intensivmediziner:innen sind als Experten für die Behandlung kritischer Zustände nicht nur für die akute Therapie verantwortlich, sondern können auch die Nachsorge früh in die richtige Richtung lenken und, in Absprache mit einem weiterversorgenden Team, interdisziplinär einen Beitrag zur Prävention von Langzeitfolgen leisten.

Die Risikofaktoren für PICS sind oftmals schwer zu quantifizieren und in ihrer Erfassung teils subjektiv [93]. Für die Zukunft ist zu erwarten, dass die klinische Einschätzung durch den Einsatz von Biomarkern und KI-basierten Prädiktionstools ergänzt und präzisiert werden kann.

Die Identifikation spezifischer Biomarker ist allerdings noch Gegenstand laufender Studien, und es ist unklar, inwieweit diese Biomarker geeignet sind, bleibende Einschränkungen vorherzusagen oder die Therapie zu steuern [94].

Ähnlich verhält es sich mit dem Einsatz von Machine-Learning-Modellen, die Daten aus elektronischen Patientenakten analysieren, um das individuelle Risiko für die Entwicklung eines PICS unter Berücksichtigung der vielschichtigen Wechselwirkungen zwischen Patientenfaktoren und der Intensivtherapie vorherzusagen [95–98]. Mittelfristig werden diese Werkzeuge Behandlungsteams zweifellos unterstützen. Ihr größter Nutzen liegt jedoch weniger in der direkten Verbesserung der Therapie, sondern in der präziseren Allokation von Ressourcen. Die unterstützenden Therapiemaßnahmen selbst sind davon unabhängig und sollten bereits heute systematisch implementiert werden.

### Entlassung von der Intensivstation – Was kommt dann?

Wenn die kritischste Phase der akuten Erkrankung überwunden ist, sollte die nächste Stufe der Versorgung, z. B. auf einer Regelversorgungsstation, mitgedacht werden. Das kurzfristige Ziel in dieser sensiblen Übergangsphase besteht darin, eine Wiederaufnahme auf die Intensivstation zu verhindern, das mittelfristige Ziel ist das zeitgerechte Erreichen der nächsten Versorgungsphase, und das langfristige Ziel ist es, individuelle Patient:innenziele in Bezug auf den Funktionsstatus zu erreichen.

Es ist von entscheidender Bedeutung, dies auch über Grenzen von Fachabteilungen hinweg zu realisieren. Jede Anstrengung, im Sinne der Patient:innen Barrieren zwischen Abteilungen abzubauen, ist wertvoll.

Zu den Voraussetzungen zum Erreichen dieser Ziele gehören die systematische interdisziplinäre Erhebung und Dokumentation des Bedarfs hinsichtlich notwendiger Therapiemaßnahmen und deren Zusammenfassung in einem umfassenden Bericht.

Für stationäre Aufenthalte i. Allg. ist das der klassische Arztbrief; für intensivstationäre Behandlungen gibt es unterschiedliche Konzepte, etwa den internen Verlegungsbericht, mit dem Intensivmediziner:innen ihren Beitrag zu einer unterbrechungsfreien Versorgung leisten können, oder aber einen vollständigen Entlassungs- oder Verlegungsbrief. In jedem Fall ist die umfassende Dokumentation des Verlaufs, inklusive Komplikationen und Organdysfunktionen, unerlässlich. Idealerweise werden, darauf aufbauend, auch spezifische Empfehlungen zur Nachsorge formuliert [99–101].

**Abb. 2:**
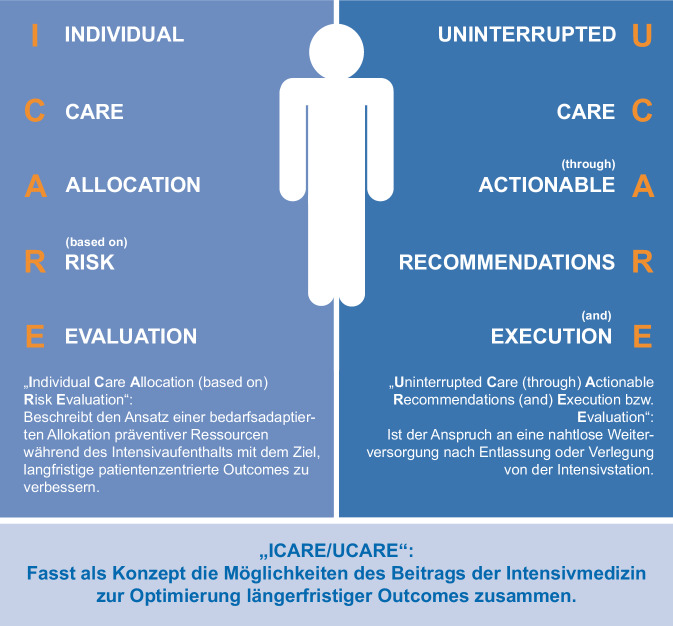
ICARE-UCARE-Prinzip des stationsübergreifenden Beitrags von Intensivmedizin zur Verbesserung der (Langzeit‑)Versorgung von Patientinnen und Patienten

Nachdem während der Intensivtherapie idealerweise zu jedem Zeitpunkt die für langfristige Outcomes entscheidenden Maßnahmen nach individuellem Bedarf der Patient:innen etabliert worden sind, gilt es, sicherzustellen, dass eine Verlegung oder Entlassung keinen Abbruch der Versorgung bedeutet (Abb. 2). Best-Practice-Modelle integrieren dabei nicht nur medizinische, sondern auch soziale, psychologische und rehabilitative Aspekte und beziehen die Patient:innen und ihre Angehörigen aktiv in die Weichenstellung für den weiteren Versorgungsweg ein.

Exzellente patient:innenzentrierte Intensivmedizin endet nicht abrupt mit der Verlegung von der Intensivstation. Sie stellt sicher, dass die weitere Versorgung so organisiert wird, dass individuelle kurz-, mittel- und langfristige Versorgungsziele erreicht werden. In enger Zusammenarbeit mit den weiterbehandelnden Ärzt:innen, den Angehörigen und den Patient:innen selbst wird so gewährleistet, dass langfristige Komplikationen minimiert und bestmögliche Ergebnisse erzielt werden können.

## Fazit für die Praxis

- Viele Patient:innen, die eine kritische Erkrankung mit intensivmedizinischer Behandlung überstehen, leiden noch lange nach der Entlassung unter anhaltenden Beschwerden.
- Intensivmediziner:innen können komplikativen Verläufen vorbeugen, indem sie zu unterschiedlichen Zeitpunkten Einschätzungen bezüglich *langfristiger* Risiken treffen und entsprechend handeln.
- Der Beitrag der Intensivmedizin beginnt mit der *individualisierten* Risikoerhebung bei Aufnahme, setzt sich in konsequenter Prävention und Behandlung akuter Organdysfunktionen fort und mündet in die konkrete Bahnung der Weiterbehandlung, inklusive *spezifischer* Nachsorgeempfehlungen.
- Komplikationen und Risikofaktoren sind *lückenlos* zu dokumentieren und im Entlassbrief klar darzustellen, um eine gezielte Weiterversorgung zu ermöglichen.
- Eine enge interdisziplinäre Zusammenarbeit über den gesamten Behandlungspfad hinweg ist notwendig, um Langzeitkomplikationen zu minimieren und die soziale wie berufliche *Reintegration* zu fördern.
